# Fluorescence and Intraoperative Ultrasound as Surgical Adjuncts for Brain Metastases Resection: What Do We Know? A Systematic Review of the Literature

**DOI:** 10.3390/cancers15072047

**Published:** 2023-03-29

**Authors:** Andrea Di Cristofori, Giovanni Carone, Alessandra Rocca, Chiara Benedetta Rui, Andrea Trezza, Giorgio Carrabba, Carlo Giussani

**Affiliations:** 1 Division of Neurosurgery, Fondazione IRCCS San Gerardo dei Tintori, Via GB Pergolesi, 20900 Monza, Italy; 2 PhD Program in Neuroscience, University of Milano-Bicocca, Piazza Ateneo Nuovo 1, 20126 Milano, Italy; 3 Department of Neurosurgery, School of Medicine, Surgery Università degli Studi di Milano-Bicocca, Piazza Ateneo Nuovo 1, 20126 Milano, Italy

**Keywords:** brainmetastasis, 5-ALA, sodium fluorescein, intraoperative ultrasound, neurosurgery

## Abstract

**Simple Summary:**

The purpose of this review stems from the need, based on the incidence of brain metastases, to investigate the roles of some intraoperative adjuncts. Surgery still plays a central role in the treatment of this disease for an optimal local control of the disease. It is therefore essential to know all the tools that can help to achieve this goal safely and with maximum results. To this end, intraoperative tumour enhancing methods and intraoperative visualisation systems were examined.

**Abstract:**

(1) Background: brain metastases (BMs) are the most common neoplasm of the central nervous system; despite the high incidence of this type of tumour, to date there is no universal consensus on the most effective treatment in patients with BMs, even if surgery still plays a primary role. Despite this, the adjunct systems that help to reach the GTR, which are well structured for other tumour forms such as ultrasound and fluorescence systems, are not yet well employed and standardised in surgical practice. The aim of this review is to provide a picture of the current state-of-art of the roles of iOUS and intraoperative fluorescence to better understand their potential roles as surgical tools. (2) Methods: to reach this goal, the PubMed database was searched using the following string as the keyword: (((Brain cerebral metastasis [MeSH Major Topic])OR (brain metastasis, [MeSH Major Topic])) AND ((5-ala, [MeSH Terms]) OR (Aminolevulinicacid [All fields]) OR (fluorescein, [MeSH Terms]) OR (contrast enhanced ultrasound [MeSH Terms])OR ((intraoperative ultrasound. [MeSH Terms]))) AND (english [Filter]) AND ((english [Filter]) AND (2010:2022 [pdat])) AND (english [Filter]). (3) Results: from our research, a total of 661 articles emerged; of these, 57 were selected. 21 of these included BMs generically as a secondary class for comparisons with gliomas, without going deeply into specific details. Therefore, for our purposes, 36 articles were considered. (4) Conclusions: with regard to BMs treatment and their surgical adjuncts, there is still much to be explored. This is mainly related to the heterogeneity of patients, the primary tumour histology and the extent of systemic disease; regardless, surgery plays a paramount role in obtaining a local disease control, and more standardised surgical protocols need to be made, with the aim of optimizing the use of the available surgical adjuncts and in order to increase the rate of GTR.

## 1. Introduction

Brain metastases (BMs) are the most common neoplasm of the central nervous system (CNS), with up to 30–40% of patients developing BMs during the course of their disease [1]. The estimated number of new cases of BMs is approximately 100,000–170,000 per year in the USA [2]. Despite the high incidence of this type of tumour, to date there is no universal consensus on the most effective treatment for patients with BM and no common guidelines have been established [3]. In the case of BM, the main goal still remains the local control, which prevents death from the neurological disease and which provides a satisfactory quality of life. To achieve these goals, multimodal management of BMs is now available and is rapidly evolving [3]. Local therapies such as whole-brain radiation therapy (WBRT), stereotactic radiosurgery (SRS) and surgical resection are combined with systemic treatments and aim to prolong the patient’s survival. Regarding the role of surgery, the European Association of Neuro-Oncology (EANO) recommends surgical removal of BMs for diagnostic purposes when no primary tumour is known or when more than one tumour can be diagnosed, and both surgery and radiation treatments for better local control of the disease [4]. Several experiences have reported the useful role of surgical resection *plus* radiation therapy for an optimal local control of BMs [5,6,7,8,9]. SRS represents a therapeutic choice for BMs with similar results to surgical resection in the case of small BMs (less than 3 cm in diameter) and it can be combined with WBRT in selected cases [10,11].

As a result, surgical resection has become the first standard treatment for brain metastases larger than 3 cm in patients with a single lesion in a safe location, especially when it causes a mass effect and when patients have a good performance status with good systemic disease control [12,13]. When BM surgery is performed, a gross total resection (GTR) can result in better local control, with a lower recurrence rate and a lower risk of leptomeningeal dissemination [14]. Consequently, achieving a GTR is of paramount importance for oncological patients; however, several authors report incomplete resection in the case of BM resection [11]. The rate of incomplete surgical resection can reach about 20% to 50% [15,16,17,18]. To date, intraoperative surgical adjuncts were introduced in recent years to reduce the rate of incomplete surgical resections in BM resection surgery.

Considering the primary role that surgery still plays in the treatment of metastases, it is imperative to shed light on those surgical adjuncts that allow surgeons to reach the GTR and that are well known and routinely used in other diseases, such as gliomas. Indeed, in recent years, the introduction of fluorescence-guided surgery [19,20,21] was shown to increase the rate of GTR in glioma surgery, especially when combined with perilesional resection and intraoperative ultrasound [22,23,24]. Nowadays, 5-aminolevulinic acid (5-ALA) and sodium fluorescein (SF) are intraoperative dyes used for surgical resection of gliomas and were also described for resection of intra-axial tumours [20,25]. The surgical dye 5-ALA is converted to protoporphyrin IX (PpIX),which accumulates more in tumour cells than in normal cells [26]. The accumulation of PpIX can be seen during surgical resection of a tumour using a special light filter called “blue light”. Tumour cells can be seen as pink, while the normal brain parenchyma remains blue. This type of intraoperative contrast helps the neurosurgeon reduce the risks of tumour remnants [26].

Sodium fluorescein (SF) is a surgical dye used in neurosurgery to resect brain tumours such as gliomas or BMs [19,27,28]. SF accumulates in specific areas of the brain where the blood-brain barrier is damaged, highlighting an area in the tumour site that can be considered to be “perilesional” [19,29,30].

Intraoperative ultrasound (iOUS) is a tool for obtaining real-time intraoperative images of the brain and of the tumour [31,32]. It has been described as a safe, fast and simple intraoperative imaging technique, capable of providing immediate feedback on the surgical cavity and possible tumour remnants [33]. This type of intraoperative imaging technique has been shown to be as reliable as intraoperative MRI in certain situations, such as paediatric brain tumours [34].

Some authors have reported their experiences with the use of intraoperative fluorescence or iOUS in cases of surgical resections of BMs, and there is clear evidence of its usefulness.

The aim of this review is to provide a picture of the current state-of-art regarding the role of iOUS and intraoperative fluorescence in cases of BM surgery and to better understand their potential roles as surgical tools for this kind of surgery.

## 2. Material and Methods

**Figure 1:**
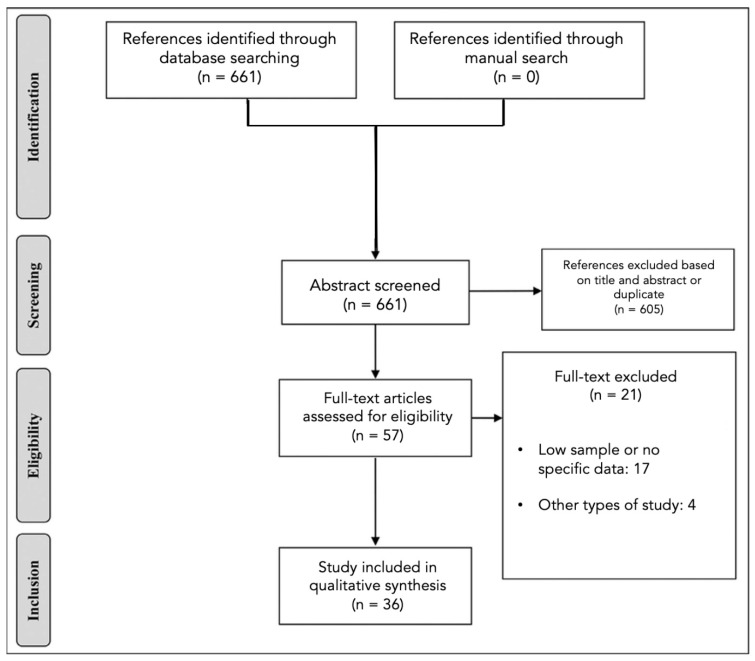
PRISMA chart.

We reviewed all the existing literature on PubMed from January 2001 to 31 December 2022. All articles were written in English, without restrictions about the paper’s publication status, according to the Preferred Reporting Items for Systematic Reviews and Meta-Analyses (PRISMA) statement [35]. The PubMed database was searched using the following string: (((cerebral metastasis [MeSH Major Topic]) OR (brain metastasis [MeSH Major Topic])) AND ((5-ala [MeSH Terms]) OR (Aminolevulinic acid [All fields]) OR (fluorescein [MeSH Terms]) OR (contrast enhanced ultrasound [MeSH Terms]) OR ((intraoperative ultrasound [MeSH Terms]))) AND (english [Filter]) AND ((english [Filter]) AND (2010:2022 [pdat])) AND (english [Filter]). All titles and abstracts were screened in order to exclude irrelevant studies. Studies dealing with fluorescence in metastases were included, with particular reference to sodium fluorescence and 5-ALA, as well as those that used iOUS with all its multi-parameter scans. Articles that did not provide precise indications about the case series analysed were excluded and studies that included BMs, but only as secondary elements of the gliomas family without going into detail, were excluded. Reviews, letters to the editor and articles not written in English were excluded. Data from the eligible works were chosen and approved through careful analysis by two blind reviewers using Rayyan software [36] (Figure 1).

## 3. Results

Although interest in intraparenchymal tumours is increasing, the literature onBMs is still scarce. Our search yielded a total of 661 articles, of which 57 were selected. Unfortunately, 21 (36.8%) of them included BMs generically as a secondary class for comparison with gliomas, without going deeply into specific details (e.g., histology of the primary tumour). Therefore, for our purposes, 36 (63.2%) articles were considered for our purposes. We found 13 out of 36 articles [15,37,38,39,40,41,42,43,44,45,46,47,48] (Table 1) dealing with 5-ALA and 10 out of 36 articles dealing with sodium fluoresceine (SF) [18,27,28,29,49,50,51,52,53,54] (Table 2). With regard to imaging modalities, on the other hand, the use of ultrasound emerges, although most articles (15 out of 36)are purely descriptive [22,55,56,57,58,59,60,61,62,63,64,65,66,67,68], (Table 3).

### 3.1. Fluorescence in Brain Metastases

In contrast to glioma surgery, fluorescence-guided surgery in BM has been poorly studied and its use is still parcelled out, since it is often tied to the school of thought of the Centre. To date, the main fluorescent systems exploited in brain tumours are 5-ALA and SF.

Significantly, among the articles selected for this review, 21/36 (58.3%) dealt with fluorescence-guided resections for BM.

### 3.2. 5-ALA in BM Resection

A total of 276 patients were analysed in the selected articles. From the literature analysis, the presence of a high fluorescence intensity of the BM varies considerably in the analysed datasets, ranging from 31% to 61% of the cases [37,44].

The largest series of patients operated on under fluorescence guidance for a BM was reported by M.A. Kamp et al. [16,37]. A study by M. A. Kamp et al. showed that the rate of 5-ALA fluorescence uptake can vary widely depending on the type of primary lesion and on the type of histology [37]. In their study, a cohort of 218 patients underwent fluorescence-guided surgery. Among their patients, 110 (50.5%) suffered from non-small cell lung cancer (NSCLC) of which 32.7% were positive to 5-ALA; 19 patients suffered from malignant melanoma, of which 21% showed 5-ALA florescence; 27 patients suffered from breast cancer and 33.3% reacted to 5-ALA; 26 patients suffered from gastrointestinal cancer, of which 15.4% reacted positively to 5-ALA; and 21 patients suffered from renal/urogenital cancer, of which 38% had positive 5-ALA fluorescence [16]. Unfortunately, no other study reported such a detailed breakdown of tumour histology and 5-ALA fluorescence.

Regarding the possible role of 5-ALA as a prognostic factor, by Kamp et al. studied 218 patients, and the median overall survival (OS)of patients with PpIX-positive BMs was 20 months, while the median OS of patients with PpIX-negative BMs was 14 months (*p* = 0.006) [16]. In their study, GTR was achieved in 123/218 patients (56.4%); however, the lack of a GTR did not show an impact on OS, while there was a trend towards recurrence in the case of 5-ALA-negativetumours. Authors also reported an extra-tumoural leakage of 5-ALA in the peritumoural tissue [47], which may indicate perilesional infiltration of the surrounding brain parenchyma [70].

### 3.3. Sodium Fluorescein in BM

A total of 381 patients were analysed in the selected articles. Ten papers reported the surgical resection of BMs using SF [Table 2].

The fluorescence rate with SF was significantly higher, reaching over 90% [29]. Okuda et al. investigated the effect of a high dose of Sodium Fluorescein (20 mg/kg) administered after durotomy without a yellow filter on the operating microscope in a sample of 36 patients, and reported a GTR rate of 86.1% [50]. Similar results were obtained by Schebesch et al. in 30 patients with aGTR rate of 83.3% [53]. Updated results from the same group showed similar GTR rates (83%) based on an additional 65 patients receiving low-dose SF (5 mg/kg dose) [27]. More recently, a retrospective study by Kofoed et al. compared the degree of resection and patient outcomes after neurosurgical treatment with either SF or white light [51]. In a total of 117 patients with first-time cerebral metastases, the results showed a statistically higher degree of resection in the SF group, with 94% having little or no measurable residual tumour compared to 84% in the non-SF group. Overall, the 1-year survival rate was significantly higher in the SF group (44.6%) compared to the non-SF group (31.1%) [48].

### 3.4. Intraoperative Ultrasound in Brain Metastases

Our search resulted in the selection of 15/36 papers dealing with BM resections under US guidance.

With regard to ultrasound, BMs are generally recognized in B-mode as distinctly hyperechoic lesions with a diffuse granular or heterogeneous aspect (e.g., a peripheral ring and a central necrosis, or nodular and cystic areas). The most common B-mode pattern includes solid components and cystic or necrotic areas of variable size with well-demarcated borders, even in the presence of surrounding oedema [67].

Contrast-enhanced US (CEUS) displays a rapid contrast enhancement; with a fast arterial phase and contrast-enhancement peak. Contrast enhancement (CE) is intense and sustained with a delayed venous phase and an irregular and heterogeneous CE pattern. CEUS shows the type of tissue (e.g., necrotic vs. vital) in detail according to the degree of CE. The arterial supply is often centripetal with many macro vessels within the lesion; their visualisation can be enhanced by MIP sequences. With such a view, CEUS can aid BM resection by identifying feeding arteries; draining veins and tumour vasculature [65].

The iOUS can also be used to recognise the nature of the tumour. In fact, BMsare usually slightly more echogenic when compared to low-grade gliomas, and due to their different mechanical properties. As proposed by some authors, the use of algorithms based on elastosonography appear to be more accurate than those from differentiating intraoperatively high-grade gliomas and BMs. A study by S. Cepeda showed that BMs had a lower Mean Tissue Elasticity (MTE) than gliomas [HGGs, 77.9 (18.9); LGGs, 91 (19.5); metastases, 103.9 (35.6); and that tumour types significantly differed [H (18.2; *p* < 0.001)] [71]. Furthermore, the Shear Wave Elastography (SWE) could be useful in detecting a residual tumour, since it seems to have a higher sensitivity when compared to the surgeons’ opinion (94% vs. 36%); However, the surgeons had a higher specificity than SWE (100% vs. 77%) [60].

With regard to the more recent possibility of navigating the ultrasound probe, there are currently no particular differences in the literature in terms of the extent of resection compared to the non-navigated form in BM. Certainly, as this is a new topic, and more in-depth studies are needed [72].

Regarding its usefulness in GTR, in a sample of 290 intra-axial tumours, including BM, iOUS, focusing on BM, showed an intended GTR rate of 87% and a false negative rate of 13% [22].

## 4. Discussion

It is well known that BMs are the most common neoplastic disease affecting the CNS [1,73]. The peculiarity and complexity of these lesions lies in their heterogeneity, which stems not only from the nature of the primary lesion, but also from the clonal sub-population that gave rise to the metastasis [74]. On the one hand, this histological variability makes their management very complex; and on the other hand, it offers a wide range of therapeutic options, the imperative of which is to personalise treatment according to the molecular/histological profile. Radiation therapy, both in the form of WBRT or SRS, represents the first therapeutic option in the case of BMs of a small size, or in cases of patients with multiple BMs [1,4]. RT is also a valuable adjuvant treatment after surgery in order to improve local control of BMs [5,6]. Although RT is one of the treatment options for BMs, some tumours may be less responsive to RT, making the management of patients with BMs very challenging.

Among the treatment options available to patients with BM, surgery remains the key to optimal local control of brain metastases [13]. Surgical treatment is often the starting point for both parenchymal decompression and the histopathological diagnosis is necessary for targeted therapy using biological samples [4]. Furthermore, surgical resection of BMs followed by WBRT appears to be the best treatment strategy in the case of patients harbouring a single BM [4]. As a consequence, achieving a GTR in this setting may affect the OS of patients. Indeed, the rate of extent of surgical resection of BMs may vary according to the reported case series and the technique used. Several authors report an incomplete resection in approximately 20% to 50% of cases [11,15,16], with an impact on OS in case of subtotal resection [70,75] due to increased risk of local recurrence and death [15].

Therefore, given the importance of surgical resection and the high rate of subtotal resection reported in literature, it is clear that it is essential to increase the efficacy of intraoperative dyes and to find other surgical adjuncts that may facilitate the visualisation of the tumour in order to obtain a GTR [3]. Integration of other technologies might also be another strategy to use.

### 4.1. 5-ALA in Neurosurgery for BMs

The heterogeneity of primary tumours giving rise to BMs is the main reason for the highly variable response to fluorescent systems. With regard to 5-ALA, the presence of the fluorescent behaviour of BMs varies considerably in the analysed datasets, ranging from 31% to 61% [37,44]. This wide variability is undoubtedly due, firstly, to the method used for fluorescence analysis, where the series with higher percentages are those that included cases with dubious or “vague” fluorescence; secondly, there is the possibility that it could be due to the histological subtype of the tumours, although the majority of BMs in all the reports are from lung NSCLC. Although there is anecdotal evidence that some histotypes respond better to 5-ALA, this has not yet been statistically confirmed [16]. To better understand which metastatic forms are most likely to acquire 5-ALA staining, individual mutations should probably be correlated to the fluorescence response. What is clear is that 5-ALA staining is related to heme group synthesis and iron metabolism [76], which may be altered in some tumours. Alteration of iron metabolism is considered a factor in more aggressive disease [77].

If there is no statistically clear-cut answer to the question of the response to 5-ALA, it is clear that subsequent discourse on its role in the extent of surgical resection or on OS is still very sketchy in the literature. In fact, it appears that PpIX-fluorescence of cerebral metastases maybe an intrinsic factor associated with a more “benign” behaviour of brain metastases and of the primary cancer. Loss of PpIX-fluorescence could reflect a more aggressive behaviour in case of brain metastases [16].

Starting from these considerations in 2016 and 2019, Kamp et al. published findings that led us to consider 5-ALA-positive staining as a prognostic marker, with a lower rate of local recurrence after surgery [37]. However, they concluded that one possible explanation for this association was that 5-ALA positive metastases were more often radically resected than 5-ALA negative metastases; on the other hand, as reported above, this aspect could also be related to the interaction between 5-ALA and the iron pathway, which was often altered in more aggressive tumours [77]. Complicating the interpretation of intra-operatory 5-ALA staining is the fact that several authors reported (in both clinical and laboratory settings) that the peritumoural parenchyma partially showed a 5-ALA positive staining [78]. This can be found regardless of the 5-ALA behaviour of the metastasis itself and it may be due to extracellular leakage of PpIX or to tumour infiltration as suggested by Masubuchi et al. in 2013 [79]. On the contrary, Schatlo et al. in 2020 reported that peritumoural 5-ALA staining is related to peritumoural neoplastic infiltration [70].

The experiences by Schatlo et al. might explain why 5-ALA positivity might lead to more unintended radical resections and possibly to a supramarginal resection that may lead to a better local control rate. Further studies are required to appreciate the oncological impact of the 5-ALA-induced fluorescence behaviour of cerebral metastases.

Taking together all the limited information available in the current literature, 5-ALA is a good candidate as a surgical adjunct for BM resection. In fact, it can help neurosurgeons to locate subcortical metastases and it can also help to guide neurosurgeons to achieve a GTR or a supramarginal resection. If used in such way, surgery with 5-ALA guidance needs to be performed in safe, non-eloquent brain areas or with intraoperative neurophysiological monitoring, as suggested for glioma surgery [80,81].

### 4.2. Sodium Fluorescence in Neurosurgery for BMs

Due to the uncertainty that characterises 5-ALA interpretation, SF has been reported as a valid alternative for fluorescence-guided surgery. Compared to 5-ALA, it is certainly less tumour-specific and its use is currently off-label (even in cases of glioma surgery), but it has a cheaper price and a positive intraoperative staining rate of more than 90% [27,49]. This high positive staining rate is due to a mechanism of action that is localised in a ruptured blood brain barrier (BBB) [46]. Indeed, SF accumulates in areas of the brain where the BBB is damaged. It is conceivable that in the future, the trend will be to operate on all the possible 5-ALA-positive BMs with 5-ALA, and to use SF only in those cases that can be 5-ALA-negative, in order to achieve a greater tumour-specific response. Some avenues to obtain the 5-ALA behaviour in advance could arise from a liquid biopsy, which would carve out the specific mutations; or even more easily from the latest frontiers of radio genomics, which are beginning to uncover a possible alteration in the intensity given by the 5-ALA response [82].

From a technical point of view, SF is a reliable tool for localization of subcortical BMs in order to a perform a more targeted surgery and to find the cleavage plane that is around the BMs. On the other hand, since SF enhances a BBB rupture, once surgical resection begins, the entire surgical field begins to turn yellow, which could represent a pitfall. SF is also a good marker in the case of BM biopsy as suggested for gliomas, and this is related to its ability to pass through the BBB when it is damaged by a malignant tumour [83].

### 4.3. Intraoperative Ultrasound in BMs

**Figure 2:**
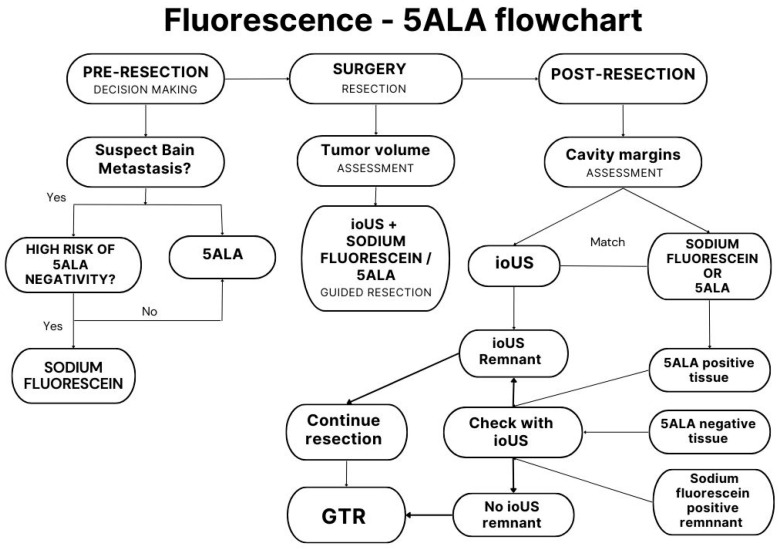
The flowchart shows the integration in the clinical setting of intraoperative fluorescence and iOUS for decision-making and resection of BMs.

Intraoperative ultrasound is now widely used as an adjuvant system to surgery that is less dependent on intrinsic variations in histology. In particular, for brain metastases, iOUS plays an important role at the beginning of the resection to localise the tumour, to understand the relationships with the major neurovascular structures, and to orient the surgeon in the surgical field. In addition, in the case of piecemeal resections, when *en-bloc* removal is not feasible, iOUS can be used to assess the extent of resection and the presence of any tumour remnants; this may overcome the limitations of dyes and may correspond to an increase in the rate of GTR. In this view, iOUS can be as effective as intraoperative MRI as suggested for paediatric brain tumours. Indeed, a recent systematic review found that iOUS had similar results to intraoperative MRI in detecting tumour remnants in the surgical field [34]. A limitation of this technique is the lack of a specific contrast agent that can specifically highlight tumour remnants. In addition, there seems to be a lack of classification of the ultrasound pattern and histology of the primary tumour. This finding would be helpful in the case of tumours of unknown origin to better understand the possible nature of the primary tumour and to better interpret the intraoperative findings in terms of 5-ALA or SF fluorescence. In this scenario, the iOUS would be linked to the surgical dyes in order to assess the extent of resection and the fluorescence seen at the surgical margins. In the graph shown in Figure 2, it is possible to understand how iOUS is interconnected with the surgical dyes and how the neurosurgeon can interpret the intraoperative findings (see Figure 2).

### 4.4. Limitations of the Studies

The main limitations of almost all the studies in the literature are characterized by a small sample size of patients included for surgery with SF, 5-ALA and iOUS. Taking into account 5-ALA, all the studies tend to consider the BMs as a unique kind of tumour, while, as considered previously, each histological BM subtype has its own biological behaviour and, as a consequence, its own uptake and metabolism for 5-ALA. Future literature should consider such features intrinsic to identifying the suspected primary tumour. On the other hand, SF does not, per se, light up perilesional infiltrative tissue. Studies performed with iOUS assistance do not report any considerations of peritumoural margins as sometimes reported for gliomas.

### 4.5. How Can We Use the Findings in the Current Literature to Improve BM Surgery?

Taking into account all the information gathered, surgical dyes are good surgical adjuncts for the resection of BMs. They allow the localisation of BMs within the brain parenchyma and they may allow the neurosurgeon to remove infiltrating perilesional regions, which may be areas of tumour recurrence after surgery. Of course, it is important to consider that perilesional fluorescence in BMs may be related to extravasation of the surgical dyes in the normal brain parenchyma, and that perilesional fluorescence can be tumour-free [47,70]. This consideration is particularly true in case of SF, due to its property of underlining BBB damaged areas, although 5-ALA has demonstrated the same behaviour in approximately 30% of cases [70].

In this setting, iOUS is helpful in determining if there are any residual tumours in the surgical field. In fact, while fluorescence can be influenced by various local and biological factors, iOUS can provide the neurosurgeon with a real-time and updated imaging of the surgical cavity. In this case, when perilesional fluorescent tissue is seen in the operating microscope or exoscope, it can be checked with iOUS, which will provide a different kind of information. Therefore, iOUS can be considered a tool for checking perilesional tissue, as well as another tool for checking for tumour remnants. Moreover, when there is any doubt about the extent of resection needed, the neurosurgeon will be able to check the surgical cavity with the iOUS, obtaining a view similar to the one with the intraoperative MRI, and reducing the risk of tumour remnants, as suggested for other types of tumours [34,64,72].

### 4.6. Perspectives on Future Studies

Our review is aimed at both clinicians and researchers involved in neuro-oncology to increase the knowledge of iOUS and fluorescence for BMs resection. Those who regularly face brain tumours during the clinical practice know that surgical adjuncts and interpretations of the intraoperative findings are key factors in avoiding any tumour remnants and in increasing patients’ OS and tumour local control.

On the other hand, our review also aims to explore perspectives for future studies to optimise BM resection. In an era of tailored and precision medicine, neurosurgery will have to improve its way of approaching each disease with dedicated, specific tools.

Firstly, starting from our review, it is clear that a specific dye for BMs is not available at this moment; and given the huge variability of tumour histotypes that can metastasize to the brain, there probably will be not a specific dye for BMs. As consequence, future studies will need to find a way to develop a dedicated intraoperative dye for each kind of BM.

Certainly, the lack of a specific contrast medium is a limitation of the treatment of this disease; it would be useful to at least investigate the existing media, and enrich the knowledge of the role of 5-ALA on the prognoses, or focus on the usefulness of fluorescence-guided supra-marginal resection in the case of positive margins. In this regard, it would be helpful to develop a radiomic model to predict 5-ALA positivity on preoperative brain MRIs. This would help the neurosurgeon to better interpret the intraoperative findings.

Another point that needs to be investigated is the correlation between fluorescent margins and perilesional histological findings with both 5-ALA and SF. Therefore, two studies would be needed: one investigating the role of supramarginal resection in BMs, and the other one investigating the finding with iOUS in cases of fluorescent margins. An in-depth study of these findings would be of great help in understanding the intra-operative findings and in tailoring surgical resections to each patient. Finally, studies taking into account the elastosonographic aspects of the surgical margins could shed some light on the infiltrative and oedematous perilesional tissue.

## 5. Conclusions

The attempt of maximal or supramarginal resection in the removal of brain metastases can be facilitated by the combination of various surgical adjuncts, including fluorescence systems and technological aids, such as iOUS, with its contrast medium. According to the available data in the literature, the lack of a specific intraoperative dye for BMs can be overcome by the use of 5-ALA or SF. On the other hand, these dyes can fail to highlight BMs. Integration of intraoperative 5-ALA and SF findings with intraoperative ultrasound imaging can increase the rate of GTR and it can lead to a better interpretation of intraoperative fluorescence.

Future studies will be devoted to finding a specific BMs dye and to finding a way to increase the rate of GTR interconnection with iOUS data.

## Figures and Tables

**Table 1:** 5-ALA articles included in the review.

Authors	Title	Aims	Findings	Limitations
Marcel A. Kamp (2016) [37]	5-ALA fluorescence of cerebral metastases and its impact for the local-in-brain progression	To analyze the oncological impact of 5-ALA fluorescence of BMsas prognostic value.	The lack of 5-ALA-induced fluorescence could increase the likelihood of local in-brain progression, but it did not have an impact on the average overall survival.	Results are limited by: (1) the retrospective study; (2) heterogeneous patient population; (3) No precise determination of EOR was performed.
Marcel A. Kamp (2011) [69]	5-Aminolevulinic acid (5-ALA)-induced fluorescence in intracerebral metastases: a retrospective study	To investigate the potential diagnostic value of 5-aminolevulinic acid (5-ALA)-induced fluorescence (5-AIF) in the resection of intracerebral metastases.	In the analyzed patients, the majority of metastases (62%) showed positive 5-ALA, indicating that 5-ALA could potentially enhance the detection of metastatic tumour tissue in the brain. Nonetheless, caution must be exercised in interpreting the presence of residual 5-AIF due to its limited specificity in detecting remaining tumour tissue.	Results are limited by: (1) the retrospective study; (2) heterogeneous patient population; (3) the presence of fluorescence was subjectively defined by one surgeon.
Jan Coburger (2014) [38]	Tumor detection with 5-aminolevulinic acid fluorescence and Gd-DTPA–enhanced intraoperative MRI at the border of contrast-enhancing lesions: a prospective study based on histopathologicalassessment	To assess whether 5-aminolevulinic acid (5-ALA) fluorescence offers any added advantages in detecting invasive tumours compared to intraoperative MRI (iMRI), including BMs.	The authors discovered that in METs, where nonenhancing lesions are prevalent, there was no added advantage of using 5-ALA over iMRI.	(1) Restricted sample size; (2) The choice of margins was subjectively defined by one surgeon.
Eric Jose Suero Molina (2013) [39]	Aquaporin-4 in glioma and metastatic tissues harboring 5-aminolevulinic acid-induced porphyrin fluorescence	Sought to use flow cytometry to determine the expression of AQPs in tumour tissue exhibiting 5-aminolevulinic acid (ALA)-induced porphyrin fluorescence, and to compare this expression to that in normal brain tissue.	No data was obtained for METs. Tissue that fluoresces under ALA showed higher AQP4 expression when compared to normal brain tissue.	(1) Restricted sample size
J.F. Cornelius (2016) [40]	Minispectrometer with handheld probe for 5-ALA basedfluorescence-guided surgery of brain tumors: Preliminary study forclinical applications	To investigate the potential of a recently developed mini-spectrometer with a handheld probe to quantify the fluorescence intensity of brain tumours based on 5-aminolevulinic acid (5-ALA), with the goal of improving fluorescence-guided neurosurgery.	The mini-spectrometer was found to be a highly sensitive tool for detecting 5-aminolevulinic acid (5-ALA)-based fluorescence in human brain tumours.	(1) Restricted sample size; (2) Depending on the number of tumour biopsies, time between resection and measurement was variable.
K. Omoto (2019) [41]	Expression of peptide transporter 1 has a positive correlation in protoporphyrin IX accumulation induced by 5-aminolevulinic acid with photodynamic detection of non-small cell lung cancer and metastatic brain tumor specimens originating from non-small cell lung cancer	To examine the mechanism underlying protoporphyrin IX fluorescence in non-small cell lung cancer (NSCLC) and in metastatic brain tumours associated with NSCLC.	The study found a positive correlation between the expression of PEPT1 and the accumulation of protoporphyrin IX induced by 5-ALA and detected through photodynamic reaction in metastatic brain tumours originating from non-small cell lung cancer (NSCLC).	(1) Immunohistochemical analysis of the clinical samples showed significant differences, but the number of clinical samples was small. (2) The current study included clinical samples from the primary lung cancer as well as the metastatic site in the brain. The specimens were not harvested simultaneously, which may pose a problem although they were collected from the same patient. The pathological and molecular status of the tumours might have been modified by treatment with chemotherapy and radiotherapy
Mikael T. Erkkilä (2020) [43]	Macroscopic fluorescence-lifetime imaging of NADH and protoporphyrin IX improves the detection and grading of 5-aminolevulinic acid-stained brain tumors	To explore the potential of macroscopic, wide-field fluorescence lifetime imaging of nicotinamide adenine dinucleotide (NADH) and protoporphyrin IX (PPIX) in selected human brain tumours.	The use of wide-field fluorescence lifetime imaging to detect nicotinamide adenine dinucleotide (NADH) and protoporphyrin IX (PPIX) can aid in distinguishing glioma tumours from metastases in the brain.	(1) Restricted sample size; (2) time between resection and measurement was variable.
David W. Roberts (2018) [44]	Red-light excitation of protoporphyrin IX fluorescence for subsurface tumor detection	To detect 5-aminolevulinic acid (ALA)-induced tumour fluorescence in brain tumours that were below the surface of the surgical field, using red-light illumination.	The study found that red-light excitation had a role inthe detection of subsurface tumours that may not be visible under conventional blue-light excitation, and couldbe performed intraoperatively.	(1) Small sample size; (2) heterogenous population.
Johannes Knipps (2019) [42]	Quantification of PpIX-fluorescence of cerebral metastases: a pilotstudy	To use a spectrometer to quantify protoporphyrin IX (PpIX)-induced fluorescence in cerebral metastases.	Approximately half of the BMs examined did not exhibit 5-ALA that was visible to the naked eye. However, over 50% of these non-fluorescent metastases showed residual 5-ALA fluorescence that could be detected and quantified using a spectrometer. Additionally, the quantified 5-ALA signal varied significantly depending on the primary tumour of the corresponding cerebral metastasis.	(1) Small sample size; (2) heterogenous population.
R. Yagi (2017) [45]	Intraoperative 5-aminolevulinic acidinducedphotodynamic diagnosis of metastatic brain tumors with histopathological analysis	To assess the utility of intraoperative fluorescence patterns and histopathological characteristics in patients with metastatic brain tumours.	The presence of peritumoural fluorescence may serve as a useful intraoperative marker for the extent of the tumour.	(1) There are possible errors in the measurement of the depth of brain invasion. The specimen slice was not always made perpendicular to the tumour surface. (2) Limited number of patients.
Serge Marbacher (2014) [46]	Use of fluorescence to guide resection or biopsy of primary brain tumors and brain metastases	To analyze the prevalence of positive 5-ALA fluorescence in a group of patients diagnosed with primary brain tumours and metastases.	5-ALA has shown promise as a tool to improve intraoperative detection of neoplastic tissue and optimize the extent of resection.	(1) Small sample size; (2) heterogenous population
Satoshi Utsuki (2007) [47]	Fluorescence-guided resection of metastatic brain tumors using a 5-aminolevulinic acid-induced protoporphyrin IX: pathological study	To identify the location where protoporphyrin IX (PPIX) is produced in human metastatic brain tumours through pathological analysis.	PPIX may be produced not only within the tumour itself, but also in the surrounding edematous tissue. It indicates that areas of peritumoural edema may also contain fluorescent tumour tissue.	(1) Sample size; (2) heterogenous population.
Ahrens L.C. (2022) [48]	5-Aminolevulinic Acid Fluorescence Indicates Perilesional Brain Infiltration in Brain Metastases	To investigate whether 5-ALA fluorescence can be a reliable indicator of metastatic brain infiltration, by analyzing the corresponding histological samples.	The study suggests that 5-ALA fluorescence accurately reflects metastatic brain infiltration, which can guide more radical resection and improve patient outcomes.	(1) Restricted sample size; (2) the choice of margins was subjectively defined by one surgeon.

**Table 2:** Sodium Fluorescein articles included in the review.

Author	Title	Aims	Conclusions	Limitations
Schebesch K.M. (2013) [49]	Sodium fluorescein–guided resection under the YELLOW560 nm surgical microscope filter in malignant brain tumor surgery—a feasibility study	Feasibility of FL in brain tumour (including METs).	A feasibility study: the authors demonstrated that the use of FL for the resection of brain tumours is safe and feasible.	(1) Sample size; (2) heterogenous population (3) no data about histology
Okuda T. (2010) [50]	Fluorescence-guided surgery of metastatic brain tumors using fluorescein sodium	To show the efficacy of using fluorescein sodium for fluorescence-guided surgery of metastatic brain tumours.	The use of fluorescein sodium in BM surgery may reduce the rate of local recurrence, and thus could help to improve the quality of life.	(1) Sample size; (2) heterogeneous patient population; (3) the presence of fluorescence was subjectively defined by one surgeon.
Kofoed M.S (2021) [51]	Fluorescein-guided resection of cerebral metastases is associated with greater tumor resection.	To find if FL correlates withBMs resection.	The use of fluorescein sodium increased the extent of resection in patients with BMs without increasing postoperative sequelae or neurological damage, regardless of the underlying primary cancer.	(1) Retrospective non-blinded approach was used; (2) the non-fluorescein group consisted ofpatients operated on from August 2014 to January 2016, while the fluorescein group was operated mainly from March 2015 to August 2018. This gives rise to a potential cohort effect.
Höhne J. (2017) [27]	Fluorescein sodium-guided resection of cerebral metastases-an update	To analyse the utility of FL in BMs resection.	FL and its YELLOW 560-nm filter are safe and feasible tools for increasing the EOR in patients with BMs.	(1) Sample size; (2) heterogenous population; (3) no data about histology.
Höhne J. (2021) [52]	Intraoperative imaging of brain tumors with fluorescein:Sconfocal laser endomicroscopy in neurosurgery. Clinicaland user experience	To report their first experiences in a clinical case series using a new confocal laser endomicroscope for brain tumours, including BMs.	The handling ergonomics and image acquisition are intuitive, and the endomicroscope allows for excellent visualization of microstructures in the surgical field. The minimally invasive technique may improve safety and clinical outcomes, indicating that this tool has the potential to advance intraparenchymal tumour surgeries, such as BMs.	(1) Small sample size; (2) subjectivity of the evaluation.
M.K. Hamamcioglu (2016) [29]	The use of the YELLOW 560 nm surgical microscope filter for sodium fluorescein-guided resection of brain tumors: Our preliminary results in a series of 28 patients	To report initial experience and preliminary results for 7 BMs patients who were operated on under Na-Fl guidance.	Na-Fl guidance with the use of a YELLOW-560 filter wassafe and effective in metastatic tumour surgery.	(1) Small sample size.
Shi-Yin Xiao (2018) [28]	Application of fluorescein sodium in breast cancer brain-metastasis surgery	To report the significance of FL-guided surgery in patients with breast cancer BMs (BCBM).	Fluorescein-guided surgery wasa simple, safe, and practical method to resect breast cancer BMs, and lead to a higher proportion of resection compared to common microsurgery. This improves the quality of life of patients with BMs.	(1) Small sample size; (2) subjective evaluation of fluorescence.
Schebesch K.M. (2015) [53]	Fluorescein sodium-guided resection of cerebral metastases—experience with the first 30 patients	To analyse the use of fluorescein sodium (FL) in patients with BMs.	FL wasa safe and practical tool for the resection of BMs.	(1) Small sample size; (2) subjective evaluation of fluorescence.
Kerschbaumer J. (2022) [18]	Mind the gap-the use of sodium fluoresceine for resection of brain metastases to improve the resection rate	To assess the effect of fluorescence-guided surgery on postoperative tumour volume and local recurrence.	The use of intraoperative fluorescence can assist neurosurgeons in achieving a more radical resection. Fluorescein appears to aid surgical resection and improve the extent of resection, ultimately reducing the risk of local recurrence.	(1) small sample size (2) possible overestimation of GTR
Okuda T. (2007) [54]	Metastatic brain tumor surgery using fluorescein sodium: technical note	To explain the use of FL in BMs with a small cohort of patients.	SF is inexpensive, highly safe, and comparatively easy to use, and does not require any special equipment.	(1) Small sample size.

**Table 3:** iOUS articles included in our review.

Author	Title	Aims	Conclusions	Limitations
Sab B. (2020) [55]	Navigated 3D Ultrasound in Brain Metastasis Surgery: Analyzing the Differences in Object Appearances in Ultrasound and Magnetic Resonance Imaging	To analyse the differences in object appearances in ultrasound (US) and magnetic resonance imaging (MRI) in 35 cases of brain metastasis.	The use of i3D US enables clear visualization of tumour boundaries and real-time imaging updates to compensate for brain shift. This can be done to a significant degree even before the dura is opened.	(1) The quality of the precalibration, respectively, the co-registration of the US probes was not sufficiently assessed for each case.
Picarelli H. (2012) [56]	Intraoperative ultrasonography for presumed brain metastases: a case series study	To find if image-guided BM resection using intraoperative ultrasound (IOUS) can lead to better surgical results.	IOUS is a useful adjunct for the removal ofBMs, but additional studies comparing its efficacy to other intraoperative exams are necessary to determine its actual value and reliability.	(1) Small sample size.
Sweeney J. (2018) [22]	Efficacy of intraoperative ultrasonography in neurosurgical tumor resection	To assess the effectiveness of IOUS in identifying gross-total resection (GTR) for brain tumour patients, including both adults and children.	Incorporating IOUS as an imaging modality may facilitate achieving a more successful GTR of brain tumours in both adult and pediatric neurosurgical patients, providing a reliable approach.	(1) Heterogenous population.
Renner C. (2005) [57]	Evaluation of intra-operative ultrasound imaging in brain tumor resection: a prospective study	To evaluate IOUS as a tool of resection control after brain tumour surgery, including BMs.	The reliability of IOUS varies depending on the type of tumour. Its use is advantageous for the resection of metastases and select high-grade gliomas. However, IOUSwasless reliableconcerning volumetric accuracy than it iseffectivein navigation and resection control.	(1) Small sample size; (2) heterogenous population.
Lindner d. (2006) [58]	Application of intraoperative 3D ultrasound during navigated tumor resection	To prove the concept of 3D ultrasound on the basis of technical and human effects.	The introduction of 3D US has increased the value of neuronavigation substantially, making it possible to update several times during surgery and minimize the problem of brain shift for intraparenchymal tumour, including BMs.	(1) Small practitioner size; (2) subjective evaluation.
Arlt F. (2016) [59]	Intraoperative 3D contrast-enhanced ultrasound (CEUS): a prospective study of 50 patients with brain tumours	To examineCEand 3D reconstructed US in brain tumour (including BMs) surgery regarding the uptake of contrast agent pre- and post-tumour resection, imaging quality and in comparison, with postoperative MRi.	3D CEUS wasa reliable intraoperative imaging modality and could improve imaging quality both for glioma tumours and BMs.	(1) Small sample size; (2) heterogenous population; (3) GTR was not objectively defined.
Cepeda S. (2022) [60]	Advantages and Limitations of Intraoperative Ultrasound Strain Elastography Applied in Brain Tumor Surgery: A Single-Center Experience	To describe the main technical aspects, usefulness, and limitations of ioUS strain elastography applied in a large case series of brain tumours, including BMs	The tumour stiffness identified through IOUS strain elastography hada potential histopathological correlation, making this rapid and versatile technique highly promising for future exploration and exploitation in the coming years.	(1) Variability of evaluation due to artifacts after dural opening; (2) variability of the frequency and amplitude of the mechanical pulsations.
Cepeda S. (2020) [61]	Comparison of intraoperative ultrasound B-mode and strain elastography for the differentiation of glioblastomas from solitary brain metastases. An automated deep learning approach for image analysis.	To compare the discriminative capacity of intraoperative ultrasound B-mode and strain elastography to differentiate GBM from BMs.	Utilizing deep learning for automated processing of ultrasound images can lead to the development of highly accurate classification algorithms that can distinguish glioblastomas from metastases using intraoperative ultrasound.	(1) The sample size is relatively small. This aspect can cause an overfitting problem and the creation of an over-optimistic predictive model. (2) Variability of elasticity threshold values and the absence of an image quality control.
Prada F. (2019) [62]	Intraoperative strain elastosonography in brain tumor surgery.	To describe the implementation of elastosonography in oncological neurosurgery for lesion discrimination and characterization.	Elastosonography allowed surgeons to understand the mechanical properties of the brain and lesions in examination and permitteda better discrimination between different tissues, compared to B-mode.	(2) Variability of evaluation due to variability of elasticity threshold values.
Unsgaard G. (2005) [63]	Ability of navigated 3D ultrasound to delineate gliomas and metastases—comparison of image interpretations with histopathology	To test the ability of a 3D US-based intraoperative imaging and navigation system to delineate gliomas and metastases in a clinical setting.	Reformatted images derived from 3D US volumes provided a clear delineation of BMs, aiding in pre-resection planning. Navigated 3D US wasequally as reliable as navigated 3D MRI in outlining metastases.	(1) Small practitioner size; (2) subjective assessment of US.
Tonnier V.M. (2001) [64]	Comparison of intraoperative MR imaging and 3D-navigated ultrasonography in the detection and resection control of lesions.	Compared two intraoperative imaging modalities:low-field MR imaging and a prototype of a 3D-navigated US in terms of imaging quality in lesion detection and intraoperative resection control.	Based on these preliminary results, intraoperative MR imaging remains superior to intraoperative ultrasonography in terms of resection control in glioma surgery and BMs surgery.	(1) Small practitioner size; (2) Subjective assessment of US for the detection of residual tumours.
Prada F. (2014) [65]	Intraoperative Contrast-Enhanced Ultrasound for BrainTumor Surgery	To provide the dynamic and continuous CEUS evaluation of a variety of brain lesions.	The findings indicate that brain metastases exhibit a nodular, heterogeneous appearance, with central necrotic areas and visible small/large vessels.	(1) Subjective evaluation of CEUS.
Wen He (2008) [66]	Intraoperative contrast-enhanced ultrasound for brain tumors	To investigate the feasibility and value of CEUS in resection for brain tumours, including BMs.	CEUS has the potential to serve as a highly valuable imaging technique, not only in defining the boundary between the tumour and the healthy brain tissue prior to resection, but also in detecting any residual tumour tissue after the initial resection.	(1) Subjective evaluation of CEUS; (2) heterogenous population.
Prada F. (2022) [67]	Multiparametric Intraoperative Ultrasound in Oncological Neurosurgery: A Pictorial Essay	To define the aspect of Brain tumours in iOUS, including BMs.	BMs are typically identifiable in: (1) B-mode hyperechoic lesions with a granular or heterogeneous appearance, including features such as a peripheral ring and central necrosis or nodule and cystic areas. The most common B-mode pattern consists of solid components and cystic or necrotic regions with well-defined borders. (2) CEUS, which reveals rapid contrast enhancement, with a fast arterial phase and contrast enhancement peak. The CE is intense and persistent. The arterial supply often follows a centripetal pattern with several macro vessels within the lesion. (3) Elastography: BMs may be stiffer (e.g., kidney, colon) or softer (e.g., lung, endometrial) than normal brain tissue, with a low mean elasticity value due in part to central necrosis.	(1) Subjective assessment of US; (2) heterogeneous pattern of BMs depending on primary tumour.
Erdogan N. (2005) [68]	Ultrasound guidance in intracranial tumor resection: correlation with postoperative magnetic resonance findings	To assess the agreement between intraoperative ultrasonography and postoperative CE MRI in detecting tumour residue.	IOUS can be a valuable adjunct. However, surgical manipulation should be minimized. In cases where tumours have preoperatively-detected cystic components near CSF-containing spaces, careful evaluation with IOUS is necessary to detect any residual cystic components. Additionally, a low-thickness echogenic rim should not be relied upon as a definitive sign of the absence of residue.	(1) Small practitioner size; (2) subjective assessment of US for the detection of the tumour.

## Data Availability

Not applicable.

## Abbreviations

Brain metastases	(BMs)
Intraoperative ultrasound	(iOUS)
Contrast enhanced US	(CEUS)
Contrast enhancement	(CE)
Gross total resection	(GTR)
Central nervous system	(CNS)
Whole-brain radiation therapy	(WBRT)
Stereotactic radiosurgery	(SRS)
Sodium Fluorescein	(SF)
